# Genomic and Metabolomic Insights into the Antimicrobial Activities and Plant-Promoting Potential of *Streptomyces olivoreticuli* YNK-FS0020

**DOI:** 10.3390/microorganisms13091964

**Published:** 2025-08-22

**Authors:** Xin Liu, Yongqin Liao, Zhufeng Shi, Te Pu, Zhuli Shi, Jianpeng Jia, Yu Wang, Feifei He, Peiwen Yang

**Affiliations:** 1 School of Agriculture, Yunnan University, Kunming 650500, China; liuxin7@stu.ynu.edu.cn (X.L.); 15969576820@139.com (Z.S.); jiajianpeng0715@163.com (J.J.); yuwang@stu.ynu.edu.cn (Y.W.); hefeifei@ynu.edu.cn (F.H.); 2 Institute of Agricultural Environment and Resources, Yunnan Academy of Agricultural Sciences, Kunming 650204, China; lyq997480@outlook.com (Y.L.); shizhfe@163.com (Z.S.); pt806232385@outlook.com (T.P.)

**Keywords:** biological control, plant-growth promotion, whole-genome sequencing

## Abstract

Streptomycetes are vital microbial resources used in agriculture and biotechnology and are diverse secondary metabolites. The *Streptomyces olivoreticuli* YNK-FS0020 strain was isolated from the rhizosphere soil in Yunnan’s Wuliangshan Forest; its functions were explored via a series of experiments and genomic analysis. Indoor assays showed that this strain inhibits seven plant pathogens (including *Fusarium oxysporum* f. sp. *cubense* Tropical Race 4) and exhibits phosphorus solubilization, siderophore production, and plant-growth promotion. Genomic analysis revealed 47 secondary metabolite biosynthetic gene clusters: 12 shared over 60% similarity with known clusters (4 exhibited 100% similarity, involving antimycin and ectoine), while 19 showed low similarity or unknown functions, indicating the strain’s potential in the development of novel compounds. Genes related to tryptophan-IAA synthesis, phosphate metabolism, and siderophore systems were annotated, while metabolomics detected indole-3-acetic acid and kitasamycin, revealing mechanisms like hormonal regulation and antimicrobial secretion. In summary, YNK-FS0020 has potential for use in plant-growth promotion and disease control, aiding agricultural microbial resource utilization.

## 1. Introduction

Soil-borne diseases, caused by soil-borne pathogens, pose a significant threat to global crop production and can result in substantial economic losses [1]. Due to the complexity of pathogenic agents, controlling soil-borne pathogens is an arduous task. These pathogens can persist in soil or plant residues for extended periods of time and can infect a wide range of hosts [2], leading to symptoms such as root rot, stunted growth, and seedling wilting in host plants. This complicates the management of crop diseases, thereby causing significant reductions in the yield and quality of important cash crops [3]. Chemical control has been the primary method for preventing these diseases. However, the improper use of chemical products can cause a series of problems, including crop residues, soil contamination, ecological imbalance, and the development of pathogen resistance [4,5]. In recent years, the concepts of ecology and sustainable development have been accepted worldwide, and biological control methods and agents have attracted increasing attention in the integrated management of soil-borne diseases [6,7]. The application of biological control agents helps combat multiple pathogens in different crops, promote plant growth, induce plant defense against pathogen attacks, and improve and maintain soil productivity [8,9].

Actinomycetes are Gram-positive bacteria with significant biosynthetic potential, capable of producing a variety of bioactive secondary metabolites [10]. The genus *Streptomyces*, the largest of the Actinomycetes, comprises over 500 species [11]. Ubiquitous in soil environments, they hold enormous agricultural application potential [12]. *Streptomyces* can synthesize diverse secondary metabolites with broad bioactivities which bring substantial benefits to plant growth and disease resistance. They produce a wide range of metabolites, such as alkaloids, terpenoids, as well as macrolides, cyclic peptides, and polyketides. Among these, antibiotics are the most prominent secondary metabolites [13,14]. Exposure to these antibiotics can hinder the biosynthesis of cell wall components [15,16], alter cell membrane permeability [17], and inhibit the activity of ribosomes, RNA, or enzymes in harmful microorganisms [18,19], thereby achieving the effect of antagonizing pathogenic microbes. Additionally, studies have shown that *Streptomyces* isolates have the potential to promote plant growth by producing indole-3-acetic acid (IAA) [20].

Whole-genome sequencing provides a foundation for comprehensively understanding the technical characteristics and safety of bacterial strains at the genetic level by generating detailed genomic data. It facilitates in-depth exploration of the antagonistic mechanisms of biocontrol strains and provides valuable information for their application. One strain of *Streptomyces olivoreticuli* YNK-FS0020 was isolated from the rhizosphere soil of Wuliangshan Forest in Dali, Yunnan Province, China. Whole-genome sequencing of YNK-FS0020 was performed using the Illumina HiSeq high-throughput sequencing platform, followed by gene prediction, functional annotation, and the prediction of secondary metabolite biosynthetic gene clusters. Combined with non-targeted metabolomics, this study aims to identify genes and metabolites related to plant-growth promotion and pathogen antagonistic activity, reveal the biocontrol mechanisms, and provide a theoretical basis for the application of *S. olivoreticuli* YNK-FS0020 in plant-growth promotion and the biological control of plant diseases.

## 2. Materials and Methods

### 2.1. Materials and Culture Media

The YNK-FS0020 strain was isolated from the rhizosphere soil of Wuliangshan Forest in Dali, Yunnan Province. The indicator pathogens involved in this study, i.e., *Fusarium oxysporum* f. sp. *cubense* Tropical Race 4 (TR4), *Fusarium solani*, *Fusarium graminearum*, *Fusarium equiseti*, *Phytophthora capsici*, *Phytophthora parasitica*, and *Rosellinia necatrix*, were provided by the Institute of Agricultural Environment and Resources, Yunnan Academy of Agricultural Sciences. The culture media used were Gause’s No. 1 liquid medium (composed of 20.0 g soluble starch, 1.0 g KNO_3_, 1.0 g KH_2_PO_4_, 0.5 g MgSO_4_·7H_2_O, 0.5 g NaCl, 0.5 g FeSO_4_·7H_2_O, dissolved in 1000 mL distilled water, with pH adjusted to 7.2 ± 0.2) and potato dextrose agar (PDA) liquid medium (composed of 200.0 g peeled potatoes, 20.0 g glucose, and 1000 mL distilled water, with pH adjusted to 5.6 ± 0.2). The solid formulations of the aforementioned media were prepared by adding 18–20 g agar to 1000 mL liquid basal medium.

### 2.2. Physiological and Biochemical Tests of Strain YNK-FS0020

A series of physiological and biochemical characterization tests was performed on the YNK-FS0020 strain, i.e., Gram staining, the citrate utilization test, fructose, lactose, mannitol and inositol utilization tests, the methyl red test, the Voges–Proskauer (V-P) test, the gelatin liquefaction test, the hydrogen sulfide (H_2_S) production test, the starch hydrolysis test, and the cellulose hydrolysis test [21]. Each physiological and biochemical test was conducted with three replicates.

### 2.3. Scanning Electron Microscopy Sample Preparation and Morphological Observation of Strain YNK-FS0020

The strain was inoculated into Gauze’s No. 1 liquid medium and cultured at 28 °C with shaking at 180 rpm for 48 h; the fermentation broth was then harvested, and bacterial cells were collected by centrifugation at 10,000 rpm and 4 °C for 10 min, followed by three washes with 0.1 mol/L phosphate-buffered saline (PBS, pH 7.2) before being fixed in 2.5% glutaraldehyde solution at 4 °C for 4 h. After rinsing with the same PBS, the cells were subjected to gradient dehydration using 30%, 50%, 70%, 90%, and 100% ethanol (15 min per concentration), dried, and coated with a 10–20 nm gold film. Finally, the surface morphological characteristics of the bacterial cells were observed and recorded using a scanning electron microscope at an accelerating voltage of 5 kV [22].

### 2.4. Antagonism Test of Strain YNK-FS0020 Against Pathogenic Fungi

Dual culture assay: The mycelia of pathogens were picked with an inoculating loop and cultured on PDA medium for 5 days. A 5-mm pathogenic cake was punched from the edge of the pathogenic colony using a puncher and placed at the center of a new PDA medium. The functional strains were inoculated at 25 mm away from the pathogenic cake in a cross shape. Plates without inoculation served as controls, with three replicates for each strain. All plates were incubated in a constant temperature incubator at 28 °C in the dark for 5–7 days. The colony diameters of pathogens were measured using the cross method, and the inhibition rates were calculated. Inhibition rate (%) = ((colony diameter of control group − colony diameter of treatment group)/(colony diameter of control group − 5 mm)) × 100.

Antimicrobial test with non-volatile substances: A single colony of strain YNK-FS0020 was picked and inoculated into PDA liquid medium, then cultured at 28 °C at 180 rpm for 48 h to prepare the fermentation broth. The fermentation broth was diluted to an OD_600_ of 1.0 and centrifuged at 10,000 rpm for 15 min at room temperature, and the supernatant was filtered through a 0.22 μm filter membrane to remove bacterial cells. Then, 200 mL of sterilized PDA solid medium was heated to melt and cooled to approximately 50 °C, and then 20 mL of the filtered fermentation supernatant was added, mixed thoroughly, and poured into plates. After the plates had solidified, pathogenic cakes were inoculated. PDA plates without added fermentation supernatant were used as controls, with three replicates for each treatment. After incubation at 28 °C for 5–7 days, the colony growth diameter was measured, and the inhibition rate was calculated [23]. Inhibition rate (%) = ((colony diameter of control group − colony diameter of treatment group)/(colony diameter of control group − 5 mm)) × 100.

### 2.5. PGP Bioactivity Assays of Strain YNK-FS0020

The nitrogen-fixing ability of the strain was determined using Ashby nitrogen-free solid medium (10 g glucose, 0.2 g K_2_HPO_4_, 0.2 g NaCl, 0.2 g MgSO_4_·H_2_O, 0.2 g K_2_SO_4_, 5 g CaCO_3_, 18 ± 2 g agar, 1000 mL distilled water) [24]. Strain YNK-FS0020 was inoculated onto this medium and cultured in a constant temperature incubator at 28 °C for 5–7 days. Its nitrogen-fixing ability was evaluated by observing the growth status of the strain and the formation of transparent zones around the colonies. The phosphate-solubilizing potential of YNK-FS0020 was qualitatively assessed using inorganic phosphorus solid medium [25]. The siderophore-producing ability of the strain was determined using CAS (Chrome Azurol S) solid detection medium [26]. After inoculation, the plates were incubated in a constant temperature incubator at 28 °C for 5–7 days, during which the bacterial growth and the formation of orange halos around the colonies were observed daily.

### 2.6. Determination of Growth-Promoting Effect of Strain YNK-FS0020 on Greenhouse Tomato Seedlings

Strain YNK-FS0020 was preserved at −80 °C. For the experiment, a bacterial loop was used to dip the bacterial suspension, and streak inoculation was performed on a newly prepared Gause’s No. 1 solid medium plate, followed by incubation at 28 °C until colonies emerged. A single colony of YNK-FS0020 was picked with a bacterial loop and inoculated into 100 mL Gause’s No. 1 liquid medium, then cultured with shaking at 28 °C at 180 rpm for 48 h to obtain the bacterial fermentation broth (with a viable count of approximately 1 × 10^9^ CFU/mL). Using sterile water irrigation (CK1) and Gause’s No. 1 liquid medium (CK2) as controls, three treatments were set up: bacterial fermentation broth (T1), 10-fold diluted bacterial fermentation broth (T2, with a viable count of approximately 1 × 10^8^ CFU/mL), and 100-fold diluted bacterial fermentation broth (T3, with a viable count of approximately 1 × 10^7^ CFU/mL).

Root irrigation treatment was applied to tomato seedlings on the 8th day after they had been transplanted into flowerpots, with 200 mL per pot. Each treatment was set with four pots of plants as biological replicates (one seedling per pot), and the experiment was independently repeated three times. After root irrigation, the tomato seedlings were placed in a greenhouse (temperature 25 °C, 16 h light/8 h dark) for growth, with sufficient soil moisture maintained during the period. Thirty days after root irrigation, the plant height, root length, stem diameter, aboveground fresh weight, and underground fresh weight were measured.

Statistical analysis was performed using SPSS 26.0 software. First, one-way analysis of variance (One-way ANOVA) was used to test the significance of differences among different treatment groups. If the ANOVA results indicated significant differences (*p* < 0.05), further pairwise comparisons between groups were conducted using the Duncan’s multiple range test, with the significance level set at *p* < 0.05. By analyzing the differences in various growth indicators (plant height, root length, stem diameter, aboveground fresh weight, and underground fresh weight) between different treatment groups and control groups, the growth-promoting effect of strain YNK-FS0020 on tomato seedlings and its concentration dependence were clarified.

### 2.7. Genome Sequencing, Annotation, and Analysis of Strain YNK-FS0020

Strain YNK-FS0020 was inoculated into Gause’s No. 1 liquid medium and cultured at 28 °C with shaking at 180 r/min for 48 h. The bacterial cells were collected after centrifuging the culture at 10,000 rpm for 15 min. Genomic DNA of the strain was extracted using the QIAamp DNA Mini Kit, and the quality and quantity of DNA were detected with a Nanodrop 2500 spectrophotometer. High-quality DNA was then used for library construction, followed by Illumina HiSeq X Ten and PacBio RSII sequencing (Majorbio Bio-Pharm Technology, Shanghai, China). Bioinformatics analysis was performed using the Majorbio Cloud Platform. Coding sequences (CDS) in the genome were predicted using Glimmer v3.02, GeneMarkS v4.3, and Prodigal v2.6.3 software. TRNAs in the genome were predicted using tRNAscan-SE v2.0.12, and rRNAs were predicted using Barrnap v0.9 software. The predicted CDS were functionally annotated against the NR, GO, KEGG, COG, CAZY, CARD, Pfam, Swiss-Prot, and TCDB databases. Gene clusters involved in secondary metabolite biosynthesis were predicted using the online antiSMASH v7.1.0 database [27].

Comparative genomic analysis was performed using various software tools. BLAST Ring Image Generator v0.95 was used to visualize genomic comparisons among the five strains [28]. OrthoVenn 3 was employed for whole-genome orthologous gene comparison [29]. In this analysis, the coding sequences (CDS) of *S. olivoreticuli* YNK-FS0020, *S. olivoreticuli* ZZ-21, *S. albireticuli* MDJK11, *S. griseus* NBC-01630, and *S. venezuelae* ATCC-10712 were used. CDS related to YNK-FS0020 were analyzed via BLAST search with specific parameters (E-value < 1 × 10^−5^, minimum alignment length percentage > 40%) [30].

### 2.8. Non-Targeted Metabolomics Analysis of Metabolites from Strain YNK-FS0020

The fermentation broth of the strain was prepared using PDA liquid medium, and samples of the prepared fermentation filtrate (centrifuged at 10,000 rpm for 15 min, with the supernatant filtered through a 0.22 μm membrane) were sent to Tsingke Biological Technology (Beijing, China) for non-targeted metabolomics analysis. The testing conditions were as follows. Chromatographic conditions: an Agilent 1290 high-performance liquid chromatography system (Agilent Technology, Waldbronn, Germany) was used, equipped with an Agilent Zorbax RRHD C18 column (Agilent Technology, New Castle, DE, USA) (100 × 3.0 mm, 1.8 μm), with the column temperature set at 35 °C, an injection volume of 1 μL, mobile phases consisting of phase A (water containing 0.1% formic acid) and phase B (acetonitrile containing 0.1% formic acid); the gradient elution program: 5% B at 0 min, 60% B at 10 min, 95% B at 13 min, 95% B maintained until 15 min, with a post-run time of 3 min and a flow rate of 0.35 mL/min; and mass spectrometric conditions: an Agilent 6546 QTOF mass spectrometer (Agilent Technology, Santa Clara, CA, USA) was used with an electrospray ionization (ESI) source, operating in both positive (ESI+) and negative (ESI−) ion modes, with capillary voltages of 4 kV (ESI+) and 3.5 kV (ESI−), respectively, a drying gas temperature and flow rate of 300 °C and 10 L/min, respectively, and a sheath gas temperature and flow rate of 350 °C and 12 L/min, respectively. The obtained data were processed for peak detection, filtering, and alignment using MS Dial v4.92, and metabolite annotation was performed against databases such as Massbank, ultimately generating a list of qualitatively and quantitatively identified substances.

## 3. Results

### 3.1. Phenotypic Characteristics and Physiological-Biochemical Properties of Strain YNK-FS0020

**Figure 1:**
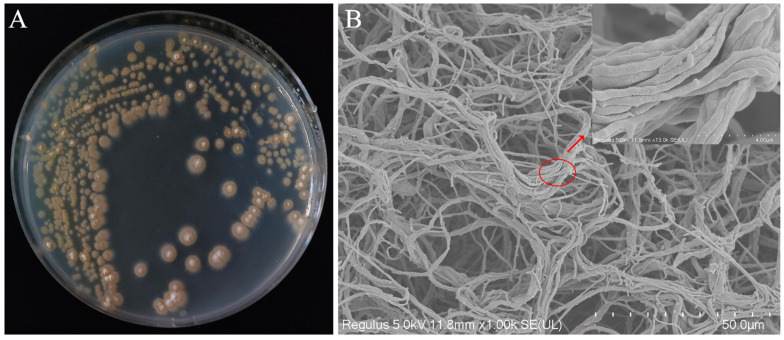
Morphological identification of strain YNK-FS0020. (**A**) Colony morphology on Gause’s No. 1 solid medium; (**B**) Scanning electron microscopy image of strain YNK-FS0020.

Morphological observations revealed that colonies of strain YNK-FS0020 on Gause’s No. 1 solid medium appeared pale yellowish-white, with a slightly elevated center and regular margins (Figure 1A). The cells of YNK-FS0020 were filamentous, either straight or curved (Figure 1B). Physiological and biochemical characterization indicated that strain YNK-FS0020 tested positive for Gram staining, gelatin liquefaction, starch hydrolysis, and cellulose hydrolysis, but negative in the methyl red test and for the Voges-Proskauer reaction and hydrogen sulfide production. Additionally, YNK-FS0020 was able to utilize citrate, fructose, lactose, and inositol as carbon sources, but not mannitol (Table S1).

### 3.2. Molecular Identification of Strain YNK-FS0020

**Figure 2:**
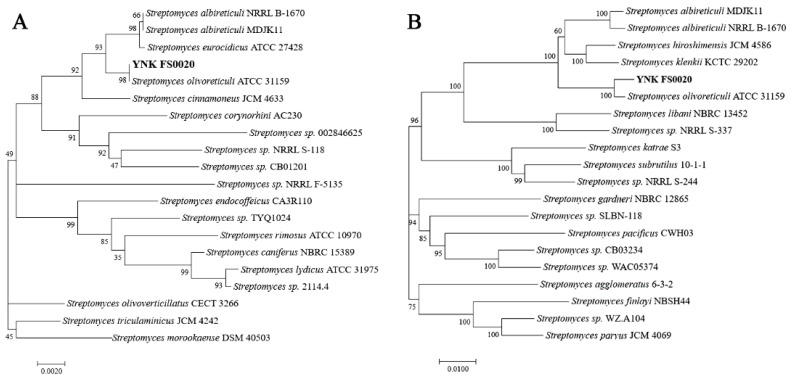
Phylogenetic tree construction based on 16S rRNA (**A**) and 31 housekeeping genes (**B**).

The 16S rRNA gene is a highly conserved sequence and is frequently used for microbial identification and classification. Phylogenetic analysis based on the 16S rRNA gene sequence showed that strain YNK-FS0020 shared 99.93% sequence similarity with *S. olivoreticuli* ATCC-31159 [31] (Figure 2A). The phylogenetic tree constructed using 31 housekeeping genes (*dnaG*, *frr*, *infC*, *nusA*, *pgk*, *pyrG*, *rplA*, *rplB*, *rplC*, *rplD*, *rplE*, *rplF*, *rplK*, *rplL*, *rplM*, *rplN*, *rplP*, *rplS*, *rplT*, *rpmA*, *rpoB*, *rpsB*, *rpsC*, *rpsE*, *rpsI*, *rpsJ*, *rpsK*, *rpsM*, *rpsS*, *smpB*, and *tsf*) also revealed that strain YNK-FS0020 had the highest homology (98.80%) with *S. olivoreticuli* ATCC-31159 (Figure 2B). Analysis of average nucleotide identity (ANI) and digital DNA-DNA hybridization (dDDH) values among 12 *Streptomyces* strains indicated that strain YNK-FS0020 shared the highest ANI (97.35%, >95%) and dDDH (76.60%, >70%) values with *S. olivoreticuli* ZZ-21, followed by *S. olivoreticuli* ATCC-31159 (Table S2). Combined with its morphological, physiological, and biochemical characteristics, strain YNK-FS0020 was identified as *S. olivoreticuli*.

### 3.3. Evaluation of Broad-Spectrum Antagonistic Activity of Strain YNK-FS0020

**Figure 3:**
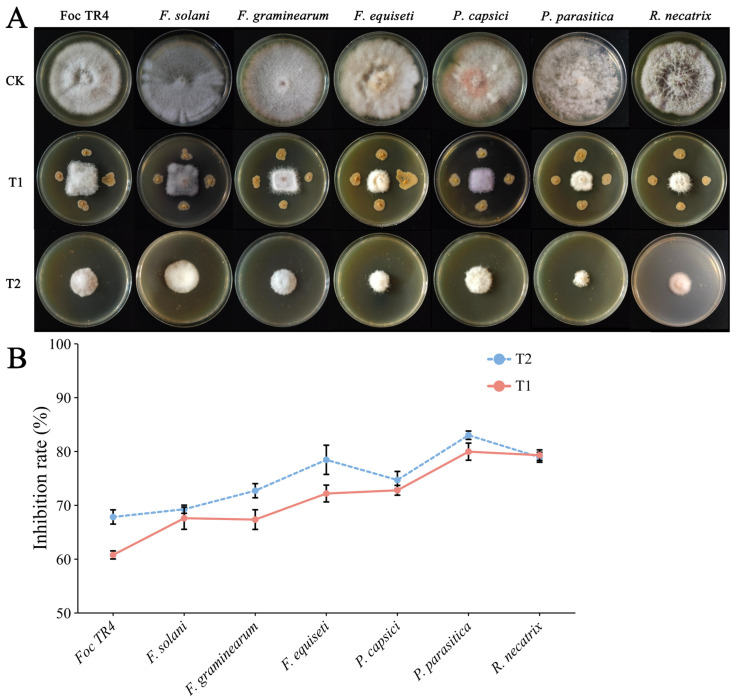
Antagonistic effect of strain YNK-FS0020 against pathogens. (**A**) Images of the antagonistic activity of strain YNK-FS0020 against various pathogens; (**B**) Inhibition rates of strain YNK-FS0020 against various pathogens. CK: Control group (pathogens cultured alone); T1: Plate confrontation assay group; T2: Non-volatile substances treatment group.

The results of the plate antagonism assay showed that strain YNK-FS0020 exhibited antagonistic activity against seven plant pathogens, namely, *F. oxysporum* f. sp. *cubense* TR4, *F. solani*, *F. graminearum*, *F. equiseti*, *P. capsici*, *P. parasitica*, and *R. necatrix* (Figure 3A). The results indicated that it had the highest inhibition rate on the mycelial growth of *P. parasitica*, reaching 79.92%, followed by *R. necatrix*, with an inhibition rate of 79.26%. The mycelial growth inhibition rates against *F. oxysporum* f. sp. *cubense* TR4, *F. solani*, *F. graminearum*, *F. equiseti*, and *P. capsici* were 60.73%, 67.56%, 67.30%, 72.14%, and 72.74%, respectively (Figure 3B).

The assay for antimicrobial activity of non-volatile substances showed that compared with the control group, the growth of the pathogens on the plates supplemented with fermentation supernatant was significantly inhibited (Figure 3A). Strain YNK-FS0020 had the strongest inhibitory effect on *P. parasitica* (82.97%), followed by *R. necatrix* (78.88%). It also exhibited obvious inhibitory effects on *F. oxysporum* f. sp. *cubense* TR4, *F. solani*, *F. graminearum*, *F. equiseti*, and *P. capsici*, with inhibition rates of 67.78%, 69.22%, 72.67%, 78.40%, and 74.66%, respectively (Figure 3B). These results indicated that the fermentation broth of strain YNK-FS0020 contained certain antimicrobial substances that could inhibit the growth of the pathogens.

### 3.4. Evaluation of PGP Bioactivity and Plant Growth-Promoting Effect of Strain YNK-FS0020

**Figure 4:**
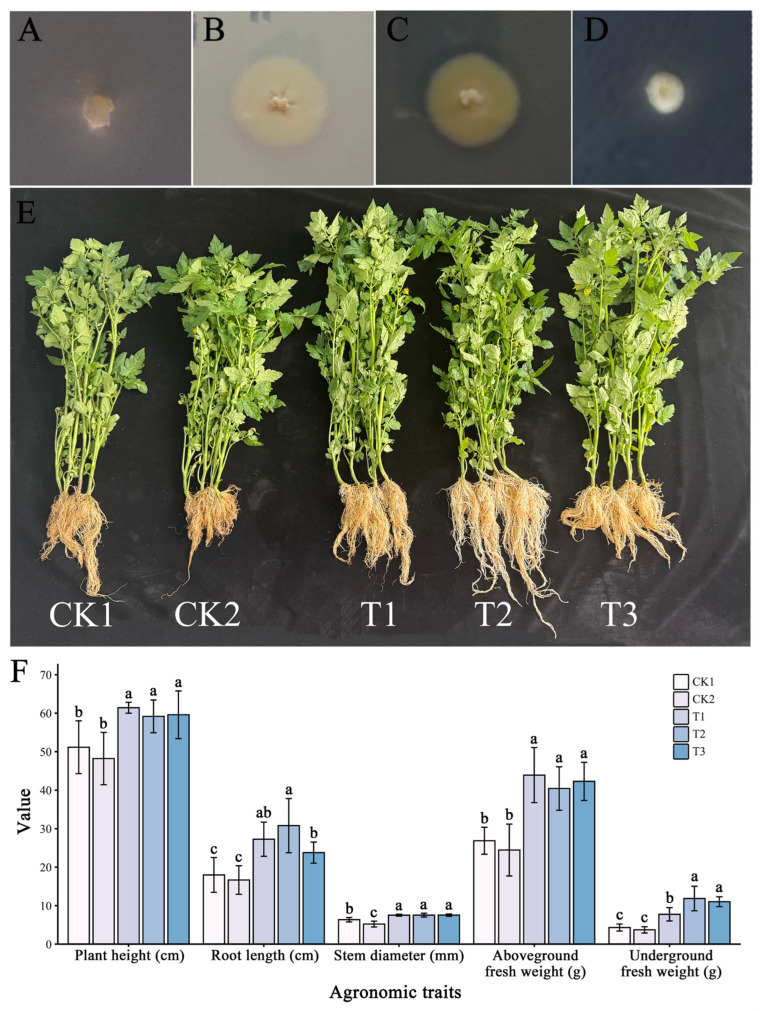
PGP bioactivity of strain YNK-FS0020 and its promoting effect on plant growth. (**A**) Nitrogen fixation; (**B**) Inorganic phosphorus solubilization; (**C**) Organic phosphorus solubilization; (**D**) Siderophore production; (**E**) Photographs of tomato plants 30 days after inoculation with YNK-FS0020; (**F**) Agronomic traits of tomato plants measured 30 days after inoculation with YNK-FS0020. Different letters indicate significant differences among treatments (Duncan’s test, *p* < 0.05). CK1: Sterile water treatment; CK2: Gause’s No. 1 liquid medium treatment; T1: Fermentation broth of strain YNK-FS0020; T2: 10-fold diluted fermentation broth of strain YNK-FS0020; T3: 100-fold diluted fermentation broth of strain YNK-FS0020.

Qualitative tests for Plant Growth-Promoting (PGP) bioactivity in this study revealed that strain YNK-FS0020 exhibited the capabilities of solubilizing inorganic (Figure 4B) and organic phosphorus (Figure 4C) and producing siderophores (Figure 4D); however, it lacked nitrogen-fixing ability (Figure 4A). Greenhouse pot experiments showed that strain YNK-FS0020 could significantly promote the growth of tomato plants. Compared with the treatment irrigated with sterile water, the tomato plants irrigated with the undiluted fermentation broth of strain YNK-FS0020 showed significant increases in plant height (20.08%), root length (51.78%), stem diameter (18.72%), aboveground fresh weight (63.65%), and underground fresh weight (80.48%) (Figure 4E,F). In addition, the agronomic traits of plants treated with 10-fold and 100-fold diluted fermentation broth were also significantly improved compared with those treated with sterile water. These results indicated that strain YNK-FS0020 has a good ability to promote plant growth and has the potential to be further developed as a plant growth-promoting agent.

### 3.5. Genome Sequencing and Analysis of Strain YNK-FS0020

**Figure 5:**
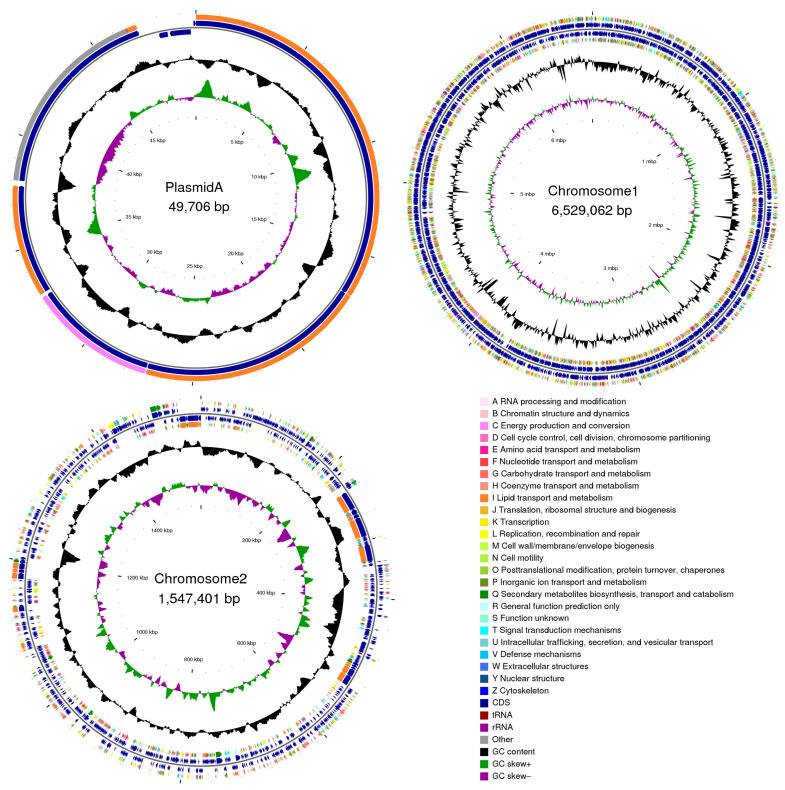
The genome circle of *S. olivoreticuli* YNK-FS0020 strain. Note: From the inside to the outside, the first circle represents the scale, the second lap represents GC Skew, the third circle represents the GC content, the fourth and seventh circles represent the COG to which each CDS belongs, and the fifth and sixth circles represent the position of CDS, tRNA, and RNA on the genome.

The whole genome of strain YNK-FS0020 was sequenced using a combined sequencing technology of the second-generation BGISEQ platform and the third-generation PacBio platform. The results showed that strain YNK-FS0020 consists of two circular chromosomes with lengths of 6,529,062 bp and 1,547,401 bp, respectively, and one plasmid with a length of 49,706 bp (Figure 5). Its GC content is 71.42%, encoding a total of 7105 genes with a gene coding rate of 88.09%. The longest sequence length is 28,503 bp, the shortest is 90 bp, and the average length of the coding genes is 1007.50 bp. There are 73 tRNA genes, belonging to 20 types in total, 21 rRNA genes, including 7 16S rRNA, 7 23S rRNA, and 7 5S rRNA genes, 31 housekeeping genes, and 86 sRNA genes with a total sequence length of 9629 bp, ranging in size from 49 to 403 bp, accounting for 0.1185% of the total genome length. There are 201 tandem repeat sequences with a total length of 40,207 bp, ranging in size from 40 to 1807 bp, accounting for 0.56% of the total genome length. Four prophages were identified in the prophage prediction, with a total sequence length of 40,732 bp; 16 genomic islands were found in the genomic island analysis. The transmembrane protein prediction results showed that 1479 protein-coding genes in the chromosomal sequences contain at least one or more transmembrane helix regions. The secreted protein prediction results indicated that 182 protein-coding genes in the chromosomal sequences contain secreted protein sequences. The genome sequencing data of strain YNK-FS0020 have been submitted to GenBank with the accession number JBMYHM000000000 (Table 1).

### 3.6. Comparative Genomic Analysis of Strain YNK-FS0020

**Figure 6:**
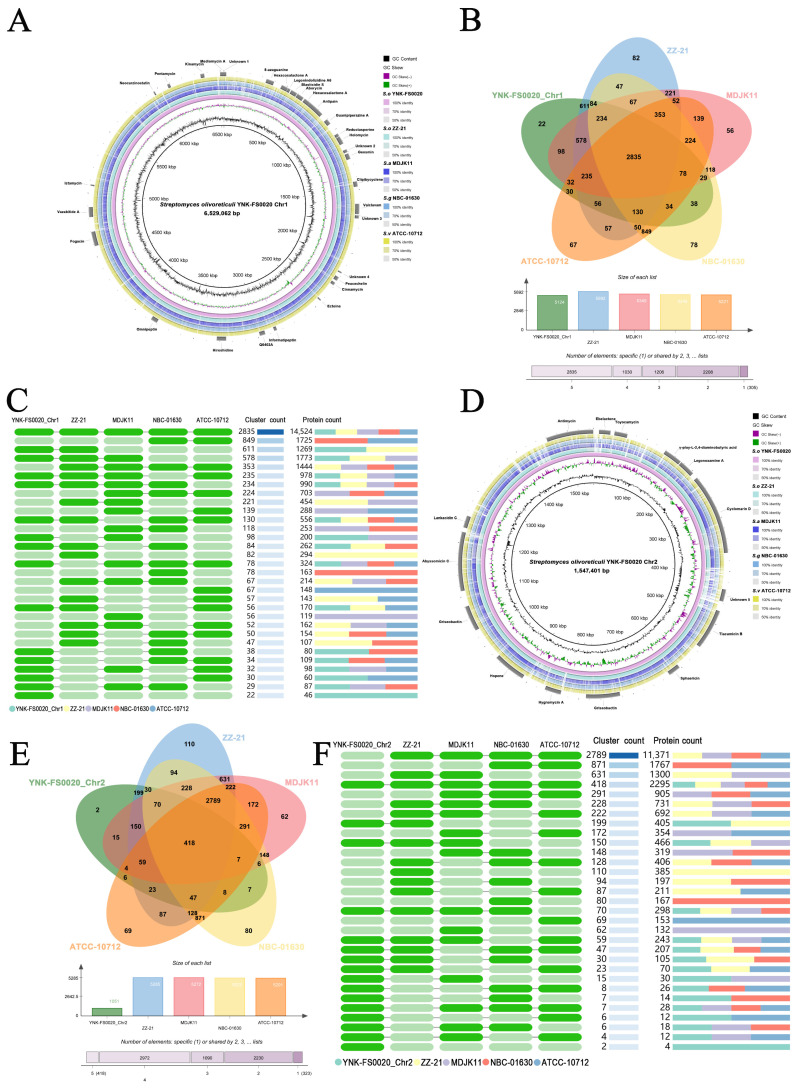
Comparative genomic analysis of *S. olivoreticuli* YNK-FS0020 chromosome with *S. olivoreticuli* ZZ-21, *S. albireticuli* MDJK11, *S. griseus* NBC-01630 and *S. venezuelae* ATCC-10712. (**A**,**D**): the comparative genomic circle map was constructed using BRIG v0.95. The features are as follows (from center to outside): circle 1 is genome size, circle 2 is GC Content, circle 3 is GC Skew, and circles 4–8 are the comparative genomic maps of *S. olivoreticuli* YNK-FS0020, *S. olivoreticuli* ZZ-21, *S. albireticuli* MDJK11, *S. griseus* NBC-01630, and *S. venezuelae* ATCC-10712, respectively; the outermost circle indicates the positions of gene clusters of predicted secondary metabolites. (**B**,**E**): Venn diagram showing the number of unique, accessory, and core genes shared in different species. The core and accessory genes are those located at the intersection of the five circles. The number of unique genes of each species is shown in each corresponding circle. The middle bar chart represents the total number of genes harbored by each individual strain. The bottommost figure shows the number of genes shared by 1, 2, 3, 4, and 5 strains, respectively. Annotation was performed using OrthoVenn3. (**C**,**F**): Presence or absence of orthologous gene clusters in different species. Dark green indicates presence, light green indicates absence.

To perform comparative genomic analyses of the chromosomal genome of *S. olivoreticuli* YNK-FS0020 with other representative *Streptomyces* strains, the complete genome sequences from the NCBI sequence database (ZZ-21, MDJK11, NBC-01630, and ATCC-10712) were analyzed using BRIG v0.95 software. The results showed that in terms of genomic structure and sequence identity, strain YNK-FS0020 has the highest whole-genome sequence similarity with *S. olivoreticuli* ZZ-21, with fewer gene variations or deletions compared to other strains (Figure 6A,D, Table S3).

In addition, pan-genomic analysis of the whole genome was conducted by comparing *S. olivoreticuli* YNK-FS0020 with *S. olivoreticuli* ZZ-21, *S. albireticuli* MDJK11, *S. griseus* NBC-01630, and *S. venezuelae* ATCC-10712. The results indicated that Chromosome 1 of *S. olivoreticuli* YNK-FS0020 shares 2835 homologous genes with the four comparison strains and contains 22 unique homologous gene clusters, encoding a total of 46 proteins (Figure 6B,C). Chromosome 2 of *S. olivoreticuli* YNK-FS0020 shares 418 homologous genes with the four comparison strains and contains 2 unique homologous gene clusters, encoding a total of 4 proteins (Figure 6E,F).

### 3.7. Genome Annotation Results of Strain YNK-FS0020

**Figure 7:**
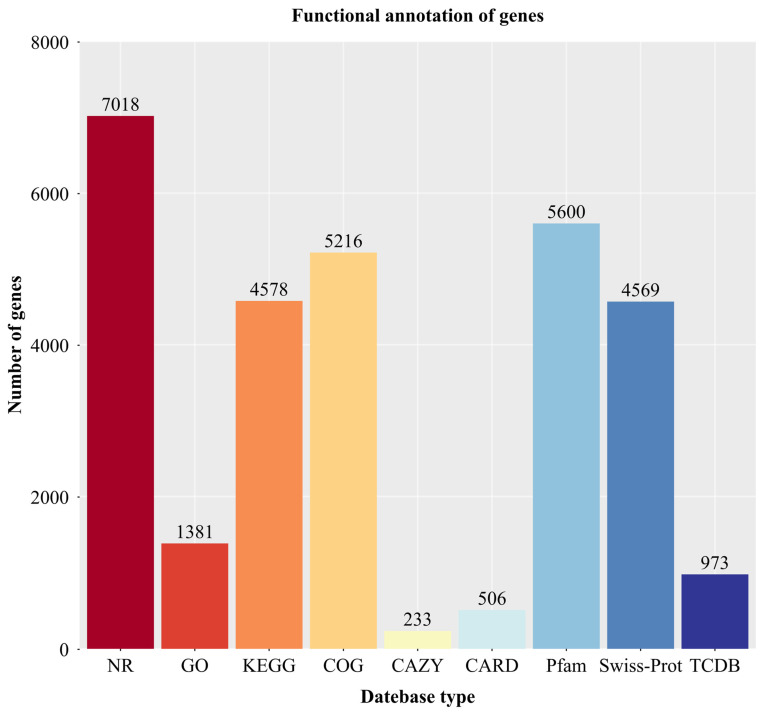
Gene functional annotation databases distribution of YNK-FS0020.

The protein sequences of the predicted genes were aligned against functional databases (NR, GO, KEGG, COG, CAZy, CARD, Pfam, Swiss-Prot, and TCDB) using Diamond (E value ≤ 1 × 10^−5^). The alignment results with the highest scores (default identity ≥ 40% and coverage ≥ 40%) were selected for annotation. The final annotation statistics are shown in Figure 7. A large number of genes were functionally annotated in the NR, Pfam, COG, KEGG, Swiss-Prot, and GO databases, with 7018, 5600, 5216, 4578, 4569, and 1381 genes, accounting for 98.78%, 78.82%, 73.41%, 64.43%, 64.31%, and 19.44% of the total genes, respectively. A total of 506 genes were annotated in the CARD database, accounting for 7.12%, and 973 genes were annotated in the TCDB database, accounting for 13.69%. The fewest genes were annotated in the CAZy database, with 233 genes, accounting for 3.28% of the total genes.

#### 3.7.1. Results of Gene Analysis in COG Database for Strain YNK-FS0020

**Figure 8:**
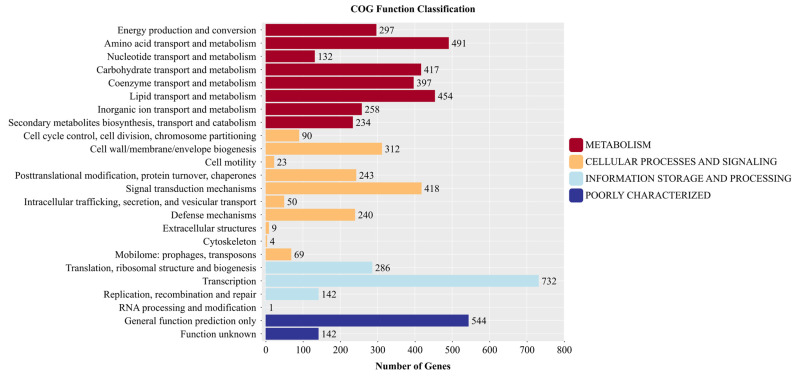
Functional annotation results of COG database in the genome of YNK-FS0020.

A total of 5216 genes from the whole-genome sequencing results of strain YNK-FS0020 were annotated using the COG database, accounting for 73.41% of all annotated genes. The results were classified into 4 major categories and 24 subcategories through multi-level classification (Figure 8). It should be noted that some genes were annotated into multiple categories. Among the 4 major categories, 2680 genes (44.78%) were classified into the metabolism category, 1458 genes (24.36%) into the cellular processes and signaling category, 1161 genes (19.40%) into the information storage and processing category, and 686 genes (11.46%) with unclear annotation characteristics. These results indicate that strain YNK-FS0020 is metabolically active, capable of providing substances, energy, and metabolic products for the bacterium. Among the 24 subcategories, the annotation results for transcription were the most abundant, with 732 genes (12.23), followed by general function prediction (544, 9.09%), amino acid transport and metabolism (491, 8.20%), lipid transport and metabolism (454, 7.59%), signal transduction mechanisms (418, 6.98%), and carbohydrate transport and metabolism (417, 6.97%). This suggests that the strain has great potential in terms of functional expression, viability, and ability to compete for nutrients with other strains.

#### 3.7.2. Results of Gene Analysis in KEGG Database for Strain YNK-FS0020

**Figure 9:**
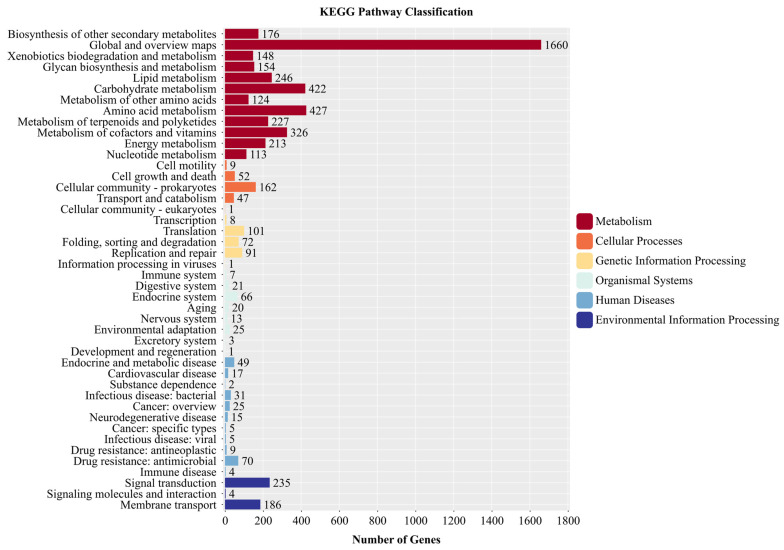
Functional annotation results of KEGG database in the genome of YNK-FS0020.

A total of 4578 genes in the genome of strain YNK-FS0020 were annotated in the KEGG database, which were classified into 6 major categories and 44 subcategories (Figure 9). Considering that some genes were assigned to multiple categories, among the 6 major categories, “Metabolism” contained the largest number of genes, with 4236 genes, accounting for 75.74%. Among the 44 subcategories, excluding 1660 annotations related to “Global and overview maps”, the most abundant ones were “Amino acid metabolism” (427 genes, 7.63%), “Carbohydrate metabolism” (422 genes, 7.55%), and “Metabolism of cofactors and vitamins” (326 genes, 5.83%).

#### 3.7.3. Results of Gene Analysis in GO Database for Strain YNK-FS0020

**Figure 10:**
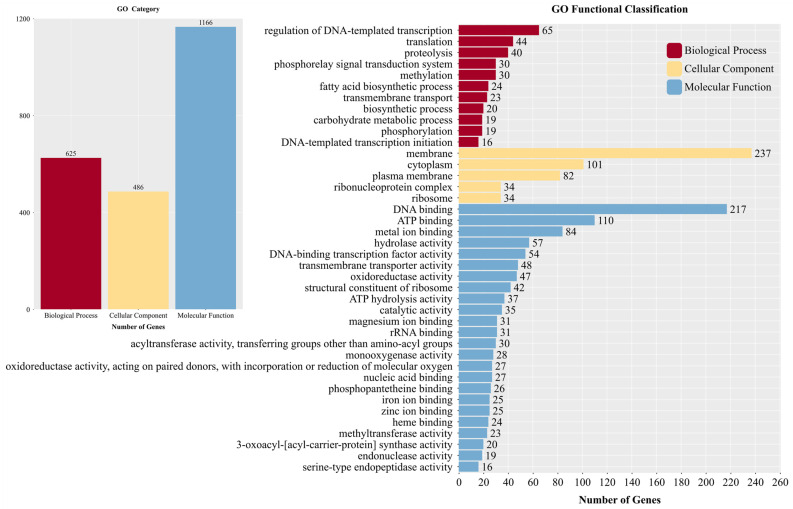
Functional annotation results of GO database in the genome of YNK-FS0020.

The amino acid sequences of strain YNK-FS0020 were aligned with the GO database and subjected to statistical analysis to determine the distribution of functional genes in the strain. A total of 1381 genes were annotated in the GO database, which categorizes proteins into three aspects: biological processes, cellular components, and molecular functions (Figure 10). There are 277, 35, and 416 branches in the biological process, cellular component, and molecular function categories, respectively, totaling 728 branches. In the biological process category, 625 genes were annotated, with the largest number of genes involved in the regulation of DNA-templated transcription, translation, and protein hydrolysis, accounting for 65, 44, and 40 genes, respectively. In the cellular component category, 486 genes were annotated, among which the genes related to membrane, cytoplasm, and plasma membrane showed the highest correlation, with 237, 101, and 82 genes, respectively. In the molecular function category, 1166 genes were annotated, with the most abundant genes involved in DNA binding, ATP binding, and metal ion binding, totaling 217, 110, and 84 genes, respectively.

#### 3.7.4. Results of Gene Analysis in CAZy Database for Strain YNK-FS0020

**Figure 11:**
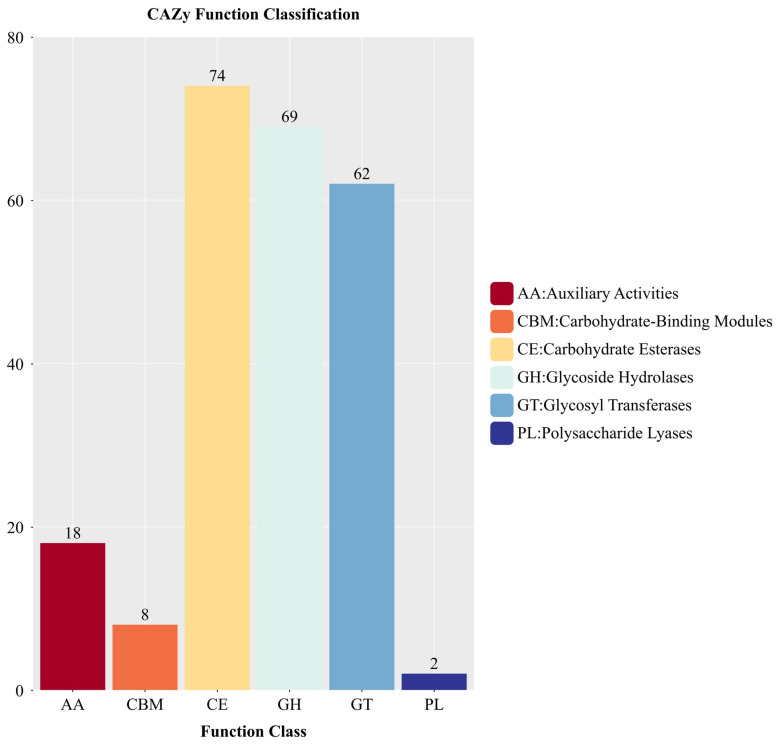
Functional annotation results of CAZy database in the genome of YNK-FS0020.

Alignment of the genome sequence with the CAZy database revealed that a total of 233 genes in the genome of strain YNK-FS0020 encode proteins with domains belonging to the CAZy family (Figure 11). These include 69 proteins from 40 glycoside hydrolase (GHs) families, 62 proteins from 14 glycosyltransferase (GTs) families, 74 proteins from 8 carbohydrate esterase (CEs) families, 18 proteins from 8 auxiliary activity (AAs) families, 8 proteins from 3 carbohydrate-binding module (CBMs) families, and 2 proteins from polysaccharide lyase (PLs) families. Among them, families related to chitin degradation, starch hydrolysis, and cellulose degradation were identified, including GH3, GH15, GH18, and GH39. Additionally, numerous genes were found in the genome of strain YNK-FS0020, which are involved in encoding endo-beta-1,4-glucanase and beta-glucosidase. These enzymes can decompose and utilize plant disease residues composed of sugars and proteins in the soil.

#### 3.7.5. Results of Gene Analysis in CARD Database for Strain YNK-FS0020

**Figure 12:**
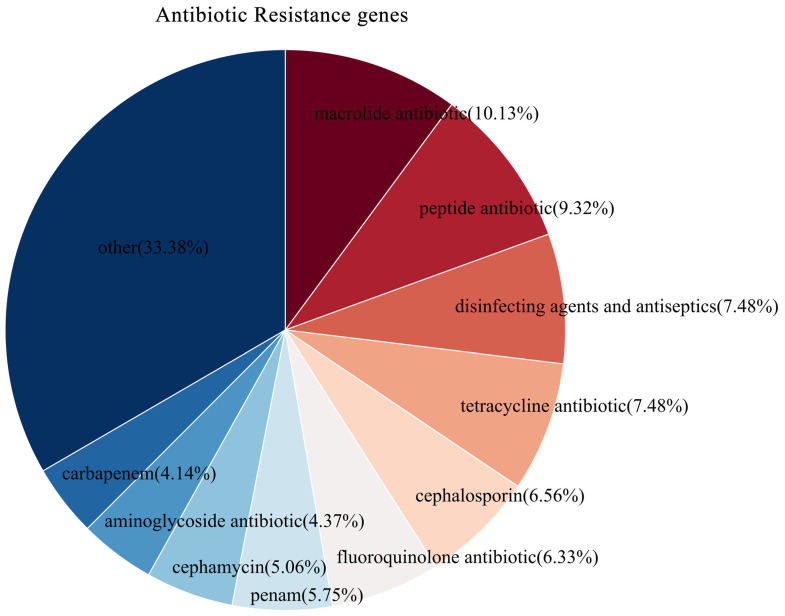
Functional annotation results of CARD database in the genome of YNK-FS0020.

Alignment of the genome of strain YNK-FS0020 with the CARD database identified a total of 506 resistance genes, with specific drug classifications shown in Figure 12. Among these, resistance genes against macrolide antibiotics (88 genes, 10.13%), peptide antibiotics (81 genes, 9.32%), disinfecting agents and antiseptics (65 genes, 7.48%), and tetracycline antibiotics (65 genes, 7.48%) were more abundant. In terms of resistance mechanisms, there were 290 antibiotic efflux genes, 51 antibiotic inactivation genes, 164 antibiotic target genes (including antibiotic biosynthesis genes), and 1 gene related to reduced permeability to antibiotics.

### 3.8. Prediction and Analysis of Secondary Metabolite Biosynthetic Gene Clusters in Strain YNK-FS0020

Prediction using antiSMASH v7.1.0 software revealed 47 secondary metabolite biosynthetic gene clusters in the genome of strain YNK-FS0020 (Table 2). Only 4 of these gene clusters showed 100% similarity to those of known compounds, namely antimycin, antipain, geosmin, and ectoine, with their biosynthetic gene cluster diagrams shown in Figure S1. The remaining 43 gene clusters exhibited similarities lower than 85%, among which 5 gene clusters had no homologous known biosynthetic gene clusters identified. Relevant studies have indicated that biosynthetic gene clusters with similarities higher than 85% are likely to be identical to their most similar known gene clusters and belong to the same known compound, while those with similarity lower than 85% may produce novel metabolites. Therefore, strain YNK-FS0020 contains numerous novel active metabolites worthy of exploration [32,33].

### 3.9. Analysis of PGP-Related Genes in Strain YNK-FS0020

Based on the annotation results from the KEGG database, genes related to IAA production, inorganic phosphorus solubilization, organic phosphorus mineralization, and siderophore production were further screened (Table 3). A complete set of tryptophan (a precursor of IAA) biosynthetic genes *trpABCDEFS* was identified in the genome of strain YNK-FS0020 (Figure S2), forming an intact biosynthetic pathway. Additionally, the *amiE* gene, which encodes amidase [EC:3.5.1.4] involved in the final step of the IAM pathway, was detected. This enzyme specifically hydrolyzes indole-3-acetamide (IAM) into IAA and ammonia, indicating that strain YNK-FS0020 has the potential to synthesize IAA without exogenous tryptophan supplementation.

The genome of YNK-FS0020 contains genes involved in phosphate transport and assimilation, such as *pstB* (ATP-binding protein), *pstAC* (permease protein), *pstS* (substrate-binding protein), *phoU* (phosphate transport system protein), and *phoR* (two-component system, OmpR family, phosphate regulon sensor histidine kinase PhoR). It also harbors *ppa* (inorganic pyrophosphatase) and phosphate metabolism regulatory genes *ppx-gppA* (exopolyphosphatase/guanosine-5′-triphosphate,3′-diphosphate pyrophosphatase). Furthermore, the YNK-FS0020 genome carries *glpQ*, a gene encoding glycerophosphodiester phosphodiesterase, which hydrolyzes glycerophosphodiesters into glycerol-3-phosphate and corresponding alcohols, participating in the catabolism of organic phosphorus compounds and converting organic phosphorus into inorganic forms utilizable by microorganisms. Genes related to pyrroloquinoline quinone (PQQ) synthesis, including *pqqC*, *pqqD*, *pqqE*, and *pqqB*, were also annotated. PQQ acts as a coenzyme for glucose dehydrogenase, which, in phosphorus-solubilizing microorganisms, collaborates with PQQ to catalyze the conversion of glucose to gluconic acid, thereby promoting the solubilization of insoluble inorganic phosphorus. Preliminary qualitative experiments also confirmed that strain YNK-FS0020 is capable of solubilizing both organic and inorganic phosphorus.

In addition, the genome of strain YNK-FS0020 contains a series of genes involved in iron uptake, transport, and siderophore biosynthesis. Among these, *entABCEF* are key genes in the enterobactin biosynthetic pathway; enterobactin is a potent iron chelator that efficiently binds environmental iron ions (especially Fe^3+^), enabling microorganisms to acquire iron nutrients in iron-deficient environments. *fepCDG* are responsible for transporting iron-bound enterobactin into cells. *EfeUOB* mediates Fe^2+^ uptake under microaerobic or anaerobic conditions and oxidizes it to Fe^3+^. *afuA* is involved in the uptake of various iron forms (e.g., free Fe^3+^ or other siderophore complexes). Preliminary experiments also confirmed the siderophore-producing ability of strain YNK-FS0020.

### 3.10. Non-Targeted Metabolomics Analysis of Strain YNK-FS0020

A non-targeted metabolomics approach was employed to detect and analyze the fermentation broth of strain YNK-FS0020, leading to the identification of 145 recognizable metabolites (Supplementary Excel S1) covering categories such as antibiotics and bacteriostatic substances, siderophores, amino acids and their derivatives, plant growth regulators, and antioxidant and antitumor active substances. Among them, antibiotic substances including kitasamycin [34], minocycline [35], and chloramphenicol [36] can exert bacteriostatic effects by inhibiting protein synthesis in pathogenic bacteria or interfering with their metabolism, facilitating the strain to gain an advantage in niche competition. Indole-3-acetic acid and its derivative (ethyl 3-indoleacetate) possess the function of regulating plant growth and may be involved in host-microbe interactions [37]. Siderophores such as deferrioxamine E [38] and ferrioxamine G1 [39] can enhance the strain’s nutrient acquisition ability by efficiently chelating iron ions in the environment, supporting its growth in iron-deficient environments. Hydroquinone [40] has antioxidant activity and can maintain cellular redox homeostasis by scavenging reactive oxygen species or chelating metal ions. Staurosporine [41], emodin [42], and mitomycin C [43] are known antitumor active substances, and their mechanisms of action may involve inhibiting tumor cell signaling pathways or inducing apoptosis. In summary, the versatility of the metabolites produced by strain YNK-FS0020 (in terms of ecological competition, nutrient utilization, biological activity regulation, etc.) indicates that it has strong comprehensive potential in environmental adaptation and biological resource development.

## 4. Discussion

Advances in genome sequencing and bioinformatics technologies have provided crucial support for microbial systematic classification and functional genomics research [44]. In this study, the taxonomic status of strain YNK-FS0020 was clarified, revealing its potential plant growth-promoting and biocontrol functions through whole-genome analysis. Phylogenetic trees constructed based on both 16S rRNA sequences and 31 housekeeping genes indicated that strain YNK-FS0020 is most closely related to *S. olivoreticuli* ATCC-31159. Further analysis of ANI and dDDH confirmed the taxonomic affiliation of strain YNK-FS0020, supporting its identification as *S. olivoreticuli*. A comparative genomic analysis revealed its unique genomic characteristics: the genome comprises two chromosomes (6.53 Mb and 1.55 Mb) and one plasmid (49.7 kb), with a total size of 8.13 Mb and 7105 coding genes. Both values are smaller than those of reference strains ZZ-21 (8.37 Mb, 7387 genes) and ATCC-31159 (8.81 Mb, 7520 genes). Notably, predictive analysis of secondary metabolite biosynthetic gene clusters (BGCs) showed that strain YNK-FS0020 contains 47 BGCs, significantly more than ZZ-21 (42) and ATCC-31159 (37). All three strains contain the ectoine biosynthetic gene cluster with 100% similarity. These results indicate that despite its relatively smaller genome, YNK-FS0020 exhibits a greater potential for secondary metabolism, i.e., it harbors more gene clusters involved in secondary metabolite biosynthesis, suggesting stronger environmental adaptability and a higher capacity for producing bioactive substances. This provides a significant theoretical basis for its application in agricultural biocontrol and plant growth promotion.

Streptomycetes, renowned for their robust capacity to produce bioactive compounds, have become one of the most studied microorganisms among prokaryotes, and they have been widely used in plant disease control in numerous studies. For instance, *S. olivoreticuli* ZZ-21 can effectively control tobacco target spot disease caused by *Rhizoctonia solani* [45]; *S.* sp. AGS-58 can efficiently inhibit the growth of *Colletotrichum siamense* on mango fruits [46]; *S. atratus* PY-1 can significantly reduce phytophthora blight caused by *P. capsici* [47]; *S.* sp. MBFA-172 [48] and H4 [49] can notably decrease the incidence of strawberry anthracnose; *S.* sp. JBS5-6 has strong inhibitory ability against spore germination and mycelial development of *F. oxysporum* f. sp. *cubense* TR4, the pathogen causing banana wilt [50]; and *S. monashensis* h114 effectively reduces the severity of citrus green mold [51]. Furthermore, Streptomycetes can produce a variety of secondary metabolites that have been applied in biopesticides, such as jinggangmycin [52] and avermectin [53]. In this study, *S. olivoreticuli* YNK-FS0020 was isolated from the rhizosphere soil of a forest in Dali, Yunnan Province. In vitro plate antagonism and non-volatile substance antifungal assays demonstrated that this strain shows good inhibitory activity against seven important soil-borne pathogens, with inhibition rates all exceeding 60%. These pathogens correspond to diseases including *F. oxysporum* f. sp. *cubense* TR4-induced banana wilt, *F. solani* root rot, *F. graminearum*-triggered wheat scab, *F. equiseti*-related root and stem diseases, *P. capsici*-associated pepper blight, *P. parasitica*-linked tobacco black shank, and *R. necatrix* fruit tree southern blight. Furthermore, this strain possesses the abilities to solubilize both organic and inorganic phosphorus, produce siderophores, and effectively promote plant growth, indicating its great application potential in plant growth promotion and biological control of soil-borne diseases.

Streptomycetes produce a diverse range of secondary metabolites through the biosynthesis of polyketides, nucleosides, peptides, and hydrolases, which can inhibit or even eliminate pathogens [54]. Additionally, Streptomycetes generate various bioactive compounds with antimicrobial properties, such as enzymes, organic acids, amino acids, immunomodulators, and vitamins [55,56,57]. A total of 47 potential biosynthetic gene clusters (BGCs) responsible for secondary metabolites were identified in the genome of strain YNK-FS0020. Among them, 12 gene clusters showed significant similarity (>60%) to known BGCs, and 4 gene clusters exhibited 100% similarity, corresponding to antimycin, antipain, geosmin, and ectoine. Antimycin compounds, a class of natural antibiotics produced by *Streptomycetes*, possess multiple biological activities, mainly including insecticidal, antifungal, antitumor, and anti-inflammatory effects [58]. Antipain is a novel antipain analog isolated from the fermentation broth of *S.* sp. MJ218-CF4; it acts as a protease inhibitor to suppress PAR signaling, thereby reducing the release of excitatory neuropeptides and inhibiting pain [59]. Ectoine, a natural compatible solute produced by halophilic microorganisms, has significant cytoprotective effects. It helps cells resist extreme environmental stresses (such as high salt, high temperature, low temperature, and desiccation) by stabilizing the structures of proteins, nucleic acids, and cell membranes [60]. Ectoine has been studied in the medical field for anti-inflammatory purposes, skin repair, and the treatment of respiratory diseases; in cosmetics, it functions as a moisturizer and antioxidant, capable of repairing UV damage and delaying skin aging; in biotechnology, it is used to enhance the stability of enzymes and cells, extending their active lifespan [61,62,63]. The identification of these gene clusters involved in the synthesis of bioactive compounds related to antimicrobial, insecticidal, and enzyme/cell stability-enhancing activities in the genome of strain YNK-FS0020 provides new insights into the disease-suppressive mechanism of this strain. Furthermore, many BGCs in strain YNK-FS0020 exhibit low similarity (<60%) to known ones, with 14 gene clusters showing less than 20% similarity and 5 gene clusters having no detectable similarity to known BGCs. This indicates that strain YNK-FS0020 carries numerous genes with potential novel functions, highlighting its significant research potential [54].

Beneficial bacterial strains promote plant growth through multiple pathways, with one core mechanism being the secretion of plant hormones (such as indole-3-acetic acid (IAA), cytokinins, and gibberellins) to directly stimulate plant growth and development [23]; for example, *Serratia marcescens* PLR promotes lateral root formation by secreting endogenously synthesized auxins and enhancing the biosynthesis of endogenous auxins in *Arabidopsis thaliana* [64]. As a direct precursor for IAA synthesis, the metabolic level of tryptophan directly regulates the efficiency of IAA synthesis [65]. In this study, the genome of strain YNK-FS0020 was annotated with 7 genes involved in tryptophan biosynthesis and 1 gene closely related to IAA synthesis. The bioavailability of phosphorus often restricts plant development; phosphate-solubilizing bacteria can improve the soil available phosphorus content by solubilizing insoluble phosphate or organic phosphorus compounds in soil, thereby indirectly promoting plant growth [23,66]. Previous studies have confirmed that *Bacillus subtilis* RS10 and *Enterobacter roggenkampii* ED5 can both solubilize soil phosphate and promote plant growth [67,68], while the genome of strain YNK-FS0020 was annotated with 13 genes related to phosphate transport, signal regulation, and metabolism. The synthesis and uptake of siderophores are important pathways for microorganisms to assist plants in acquiring iron and inhibiting pathogens, as siderophores can improve iron bioavailability by chelating environmental iron ions and inhibit pathogen growth by competing for iron sources, thereby maintaining plant health [69]. Five genes related to siderophore synthesis and 7 genes related to siderophore uptake were identified in the genome of strain YNK-FS0020. In addition, non-targeted metabolomics analysis further confirmed that the fermentation broth of strain YNK-FS0020 contains various functional metabolites, including kitasamycin and minocycline with antibacterial activity, indole-3-acetic acid that promotes plant growth, hydroquinone with antioxidant activity, and staurosporine with potential antitumor activity. In summary, strain YNK-FS0020 has comprehensive potential in terms of promoting plant growth and maintaining plant health through multiple mechanisms such as the tryptophan-IAA synthesis pathway, phosphorus-solubilizing metabolism, siderophore-mediated iron nutrient supply, and secretion of antibacterial substances, providing a theoretical basis for its application in agricultural production.

In summary, the results of this study indicate that strain YNK-FS0020 exhibits significant in vitro inhibitory activity against various soil-borne pathogens and harbors potential plant growth-promoting traits such as IAA production, phosphate solubilization, and siderophore production. Additionally, the fermentation broth of this strain contains multiple functional metabolites including indole-3-acetic acid and kitasamycin. These characteristics collectively demonstrate that the strain has potential application value in the biological control of soil-borne diseases. However, current research still has several important limitations. Firstly, key functional parameters such as IAA production, phosphate solubilization efficiency, and siderophore production capacity have not yet been quantitatively characterized. Secondly, there is a lack of in vivo validation data from plant inoculation experiments to evaluate its actual control efficacy. The lack of such critical data constrains the accurate assessment of the strain’s application potential.

## 5. Conclusions

Strain YNK-FS0020, isolated from the rhizosphere soil of Wuliang Mountain forest in Dali, was identified as *S. olivoreticuli* through whole-genome sequencing. This strain not only exhibits strong antagonistic activity against various plant pathogens but also possesses comprehensive biological activities such as phosphorus solubilization (both organic and inorganic phosphorus), siderophore production, and plant growth promotion, demonstrating broad application potential in the biological control of plant diseases and crop growth promotion. Genomic analysis revealed that it contains 47 secondary metabolite biosynthetic gene clusters (BGCs), including those with known functions such as antimycin and ectoine, as well as a large number of BGCs with low similarity or unknown functions, confirming its abundant secondary metabolic potential. At the mechanistic level, the strain exerts a synergistic effect through multiple pathways. In summary, YNK-FS0020 is a multifunctional microbial resource with both application value and research prospects. Its genomic and metabolomic data provide a molecular basis for elucidating biological functions. In the future, heterologous expression, gene editing, and other technologies could be used to further explore the synthesis mechanism of active substances, promoting its industrial application.

## Figures and Tables

**Table 1:** Genomic features of strain YNK-FS0020.

Characteristics	Value
Genome size (bp)	8,126,169
Chromosome1 size (bp)	6,529,062
Chromosome2 size (bp)	1,547,401
PlasmidA	49,706
GC content (%)	71.42
Protein-coding genes (CDS)	7105
tRNA	73
rRNA (5S, 16S, 23S)	21
House-keeping gene	31
sRNA	86
Tandem repeat	201
Prophge	4
Genomic islands	16
transmembrane protein-coding genes	1479
secretory protein-coding genes	182
GeneBank accession number	JBMYHM000000000

**Table 2:** Secondary metabolite biosynthetic gene clusters in strain YNK-FS0020 predicted by antiSMASH.

Region No.	Nucleotide Length (bp)	Gene Cluster Type	Most Similar Known Gene Cluster	Similarity (%)
PlasmidA				
Region 1.1	49,706	T1PKS	Nocamycin	18
Chromosome2				
Region 2.1	21,281	terpene	Ebelactone	5
Region 2.2	23,959	butyrolactone, nucleoside	Toyocamycin	30
Region 2.3	34,047	NAPAA	γ-ploy-L-2,4-diaminobutyric acid	50
Region 2.4	29,785	NI-siderophore	Legonoxamine A	66
Region 2.5	150,874	T1PKS, NRPS, betalactone, indole	Cyclomarin D	73
Region 2.6	22,697	lanthipeptide-class-iv	-	-
Region 2.7	75,380	T1PKS	Tiacumicin B	19
Region 2.8	22,580	lassopeptide	Sphaericin	50
Region 2.9	63,001	NRPS, terpene	Griseobactin	61
Region 2.10	30,123	2dos	Hygromycin A	51
Region 2.11	60,453	RiPP-like, terpene, NAPAA	Hopene	76
Region 2.12	47,461	NRP-metallophore, NRPS	Griseobactin	38
Region 2.13	110,141	NRPS, indole, T1PKS	Abyssomicin C	21
Region 2.14	22,073	redox-cofactor	Lankacidin C	20
Region 2.15	87,502	NRPS, T1PKS	Antimycin	100
Chromosome1				
Region 3.1	17,816	lassopeptide	-	-
Region 3.2	61,588	hglE-KS, NI-siderophore	8-azaguanine	54
Region 3.3	51,288	T1PKS, hglE-KS	Hexacosalactone A	13
Region 3.4	21,188	terpene	Legonindolizidine A6	12
Region 3.5	50,711	NRPS, terpene, NRPS-like	Blasticidin S	32
Region 3.6	55,001	hglE-KS, hydrogen-cyanide	Aborycin	21
Region 3.7	51,342	hglE-KS, T1PKS	Hexacosalactone A	13
Region 3.8	128,139	NRPS, NRPS-like, T1PKS, terpene	Antipain	100
Region 3.9	43,126	NRPS-like	Guanipiperazine A	80
Region 3.10	23,255	indole	Reductasporine	66
Region 3.11	61,661	NRPS, terpene	Holomycin	15
Region 3.12	22,727	lanthipeptide-class-iv	-	-
Region 3.13	46,396	terpene, NAPAA	Geosmin	100
Region 3.14	41,056	T3PKS	Clipibycyclene	13
Region 3.15	80,779	T1PKS, PKS-like, NRPS, blactam	Valclavam	57
Region 3.16	21,188	terpene	-	-
Region 3.17	10,852	RiPP-like	-	-
Region 3.18	31,669	NI-siderophore	Peucechelin	20
Region 3.19	23,225	lanthipeptide-class-ii	Cinnamycin	52
Region 3.20	10,399	ectoine	Ectoine	100
Region 3.21	22,673	lanthipeptide-class-iii	Informatipeptin	42
Region 3.22	43,771	T1PKS	Q6402A	7
Region 3.23	73,129	Melanin, T2PKS	Hiroshidine	34
Region 3.24	51,591	NRPS	Omnipeptin	17
Region 3.25	113,798	T3PKS, T2PKS, butyrolactone, NRPS, NRPS-like	Fogacin	40
Region 3.26	78,902	NRPS-like, NRPS, T3PKS, lassopeptide	Vazabitide A	76
Region 3.27	10,444	melanin	Istamycin	2
Region 3.28	10,972	butyrolactone	Neocarzinostatin	4
Region 3.29	21,074	terpene	Pentamycin	13
Region 3.30	29,914	NI-siderophore	Kinamycin	19
Region 3.31	30,319	T1PKS	Mediomycin A	72

**Table 3:** Predicted PGP-related genes in the genome of strain YNK-FS0020.

PGP Activities	Gene Name	KO ID	KO Description
IAA production	*trpC*	K01609	indole-3-glycerol phosphate synthase [EC:4.1.1.48]
	*trpA*	K01695	tryptophan synthase alpha chain [EC:4.2.1.20]
	*trpE*	K01657	anthranilate synthase component I [EC:4.1.3.27]
	*trpD*	K00766	anthranilate phosphoribosyltransferase [EC:2.4.2.18]
	*trpS*	K01867	tryptophanyl-tRNA synthetase [EC:6.1.1.2]
	*trpF*	K01817	phosphoribosylanthranilate isomerase [EC:5.3.1.24]
	*amiE*	K01426	amidase [EC:3.5.1.4]
	*trpB*	K01696	tryptophan synthase beta chain [EC:4.2.1.20]
Phosphate metabolism	*pstB*	K02036	phosphate transport system ATP-binding protein [EC:7.3.2.1]
	*pstA*	K02038	phosphate transport system permease protein
	*pstC*	K02037	phosphate transport system permease protein
	*glpQ*	K01126	glycerophosphoryl diester phosphodiesterase [EC:3.1.4.46]
	*phoR*	K07636	two-component system, OmpR family, phosphate regulon sensor histidine kinase PhoR [EC:2.7.13.3]
	*ppx-gppA*	K01524	exopolyphosphatase/guanosine-5′-triphosphate,3′-diphosphate pyrophosphatase [EC:3.6.1.11 3.6.1.40]
	*ppa*	K01507	inorganic pyrophosphatase [EC:3.6.1.1]
	*pstS*	K02040	phosphate transport system substrate-binding protein
	*phoU*	K02039	phosphate transport system protein
	*pqqC*	K06137	pyrroloquinoline-quinone synthase [EC:1.3.3.11]
	*pqqD*	K06138	pyrroloquinoline quinone biosynthesis protein D
	*pqqE*	K06139	PqqA peptide cyclase [EC:1.21.98.4]
	*pqqB*	K06136	pyrroloquinoline quinone biosynthesis protein B
Siderophore	*entF*	K02364	L-serine---[L-seryl-carrier protein] ligase [EC:6.3.2.14 6.2.1.72]
	*entB*	K01252	bifunctional isochorismate lyase/aryl carrier protein [EC:3.3.2.1 6.3.2.14]
	*entE*	K02363	2,3-dihydroxybenzoate---[aryl-carrier protein] ligase [EC:6.3.2.14 6.2.1.71]
	*entC*	K02361	isochorismate synthase [EC:5.4.4.2]
	*entA*	K00216	2,3-dihydro-2,3-dihydroxybenzoate dehydrogenase [EC:1.3.1.28]
	*efeB*	K16301	deferrochelatase/peroxidase EfeB [EC:1.11.1.-]
	*efeU*	K07243	high-affinity iron transporter
	*efeO*	K07224	iron uptake system component EfeO
	*afuA*	K02012	iron(III) transport system substrate-binding protein
	*fepD*	K23186	iron-siderophore transport system permease protein
	*fepG*	K23187	iron-siderophore transport system permease protein
	*fepC*	K23188	iron-siderophore transport system ATP-binding protein [EC:7.2.2.17 7.2.2.-]

## Data Availability

Genome sequences were deposited in the GenBank database under accession number JBMYHM000000000 (https://www.ncbi.nlm.nih.gov/nuccore/JBMYHM000000000.1/, accessed on 16 March 2025).

## Supplementary Materials

The following supporting information can be downloaded at: https://www.mdpi.com/article/10.3390/microorganisms13091964/s1, Supplementary Excel S1: Compound annotation table for non-targeted metabolomics of YNK-FS0020 fermentation broth; Figure S1: Known secondary metabolite biosynthetic gene clusters (with 100% similarity) in the genome of strain YNK-FS0020 predicted by antiSMASH; Figure S2: Tryptophan biosynthesis pathway of strain YNK-FS0020; Table S1: Physiological and biochemical characteristics of strain YNK-FS0020; Table S2: ANI and dDDH values between the genome of strain YNK-FS0020 and reference genomes of the genus *Streptomyces*; Table S3: Genomic comparison among *S. olivoreticuli* YNK-FS0020, *S. olivoreticuli* ZZ-21, *S. albireticuli* MDJK11, *S. griseus* NBC-01630, and *S. venezuelae* ATCC-10712.
