# Supplementary material for: Genomic and Metabolomic Insights into the Antimicrobial Activities and Plant-Promoting Potential of Streptomyces olivoreticuli YNK-FS0020
Source: Microorganisms. 2025 Aug 22;13(9):1964. doi: 10.3390/microorganisms13091964 (PMC12472026; doi:10.3390/microorganisms13091964)
Supplement: Supplementary file 1 [file microorganisms-13-01964-s001.zip › microorganisms-3799909-SI.pdf]

Article

Genomic and Metabolomic Insights into the Antimicrobial Activities and Plant-Promoting Potential of *Streptomyces olivoreticuli* YNK-FS0020

Xin Liu [1,2], Yongqin Liao [2], Zhufeng Shi [2], Te Pu [2], Zhuli Shi [1,2], Jianpeng Jia [1,2], Yu Wang [1,2], Feifei He [1] and Peiwen Yang [2,*]

[1] School of Agriculture, Yunnan University, Kunming 650500, China; liuxin7@stu.ynu.edu.cn (X.L.); 15969576820@139.com (Z.S.); jiajianpeng0715@163.com (J.J.); yuwang@stu.ynu.edu.cn (Y.W.); hefeifei@ynu.edu.cn (F.H.)

[2] Institute of Agricultural Environment and Resources, Yunnan Academy of Agricultural Sciences, Kunming 650204, China; lyq997480@outlook.com (Y.L.); shizhfe@163.com (Z.S.); pt806232385@outlook.com (T.P.)

* Correspondence: pwyang2000@126.com

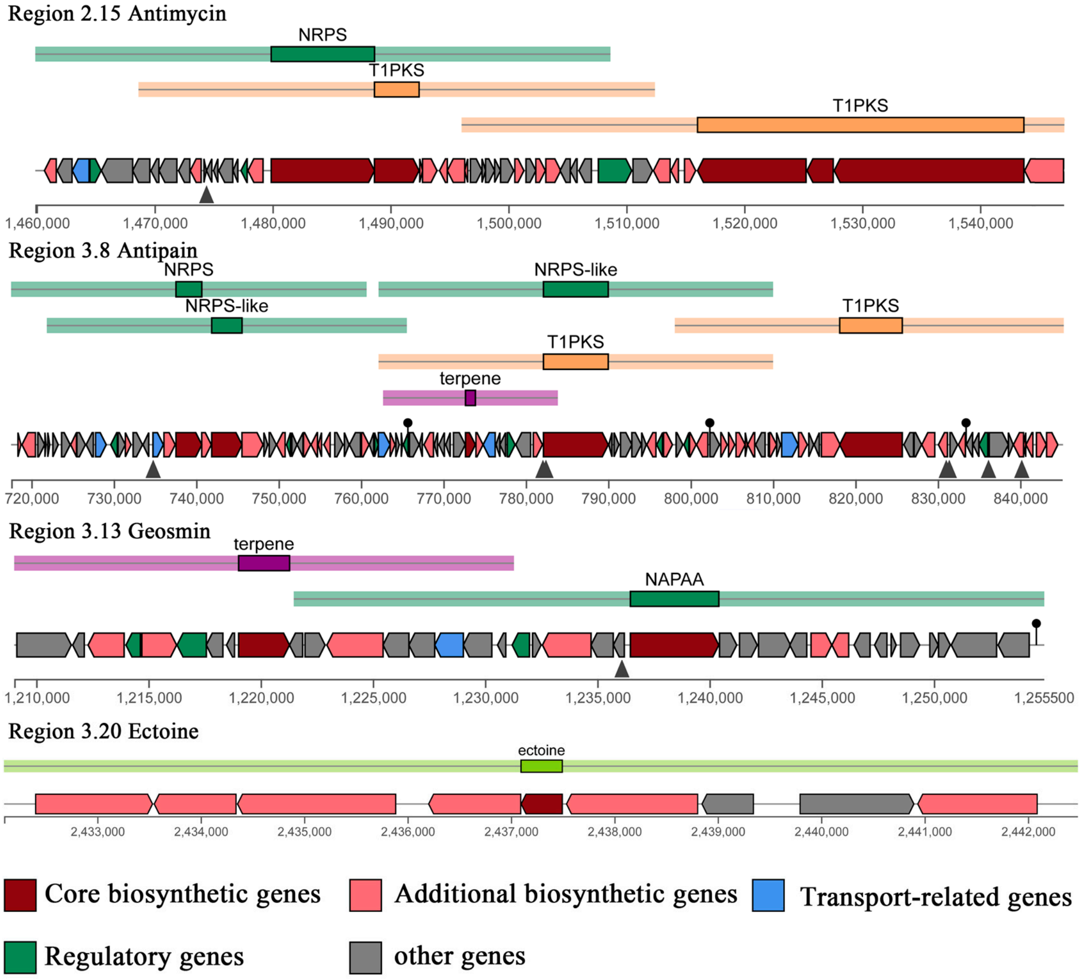

Figure S1. Known secondary metabolite biosynthetic gene clusters (with 100% similarity) in the genome of strain YNK-FS0020 predicted by antiSMASH.

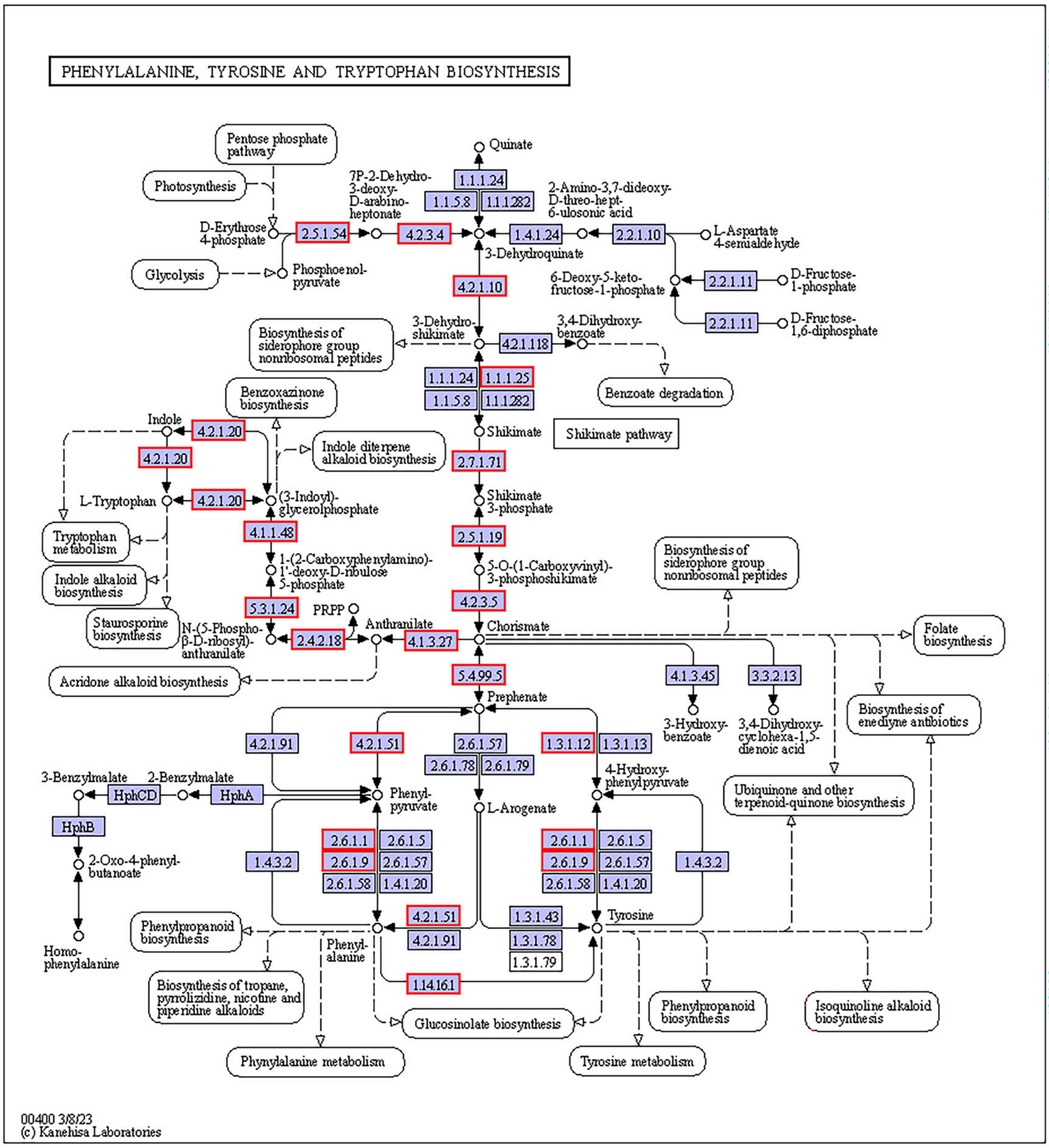

Figure S2. Tryptophan biosynthesis pathway of strain YNK-FS0020.

Table S1. Physiological and biochemical characteristics of strain YNK-FS0020.

Physiological and biochemical reaction	Results	Physiological and biochemical reaction	Results
Gram staining	+	Methyl red test	-
Citrate utilization	+	V-P reaction	-
Fructose utilization	+	Gelatin liquefaction	+
Lactose utilization	+	H_2S production	-
Mannitol utilization	-	Starch hydrolysis	+
Inositol utilization	+	Cellulose hydrolysis	+

"+", Positive reaction; "-", Negative reaction.

Table S2. ANI and dDDH values between the genome of strain YNK-FS0020 and reference genomes of the genus *Streptomyces*.

Reference genome	Accession number	ANI/%	dDDH/%
Streptomyces olivoreticuli ZZ-21	CP129915.1	97.35	76.60
Streptomyces olivoreticuli ATCC-31159	CP031455.1	95.83	64.70
Streptomyces albireticuli MDJK11	CP021744.1	84.73	29.00
Streptomyces sp. TYQ1024	CP092051.1	81.21	25.00
Streptomyces rimosus ATCC-10970	CP023688.1	79.39	23.40
Streptomyces lydicus M01	CP086217.1	79.27	23.30
Streptomyces lydicus GS93	CP019457.1	79.24	23.30
Streptomyces caniferus NBC-00314	CP108029.1	79.15	23.50
Streptomyces libani DSM-40555	CP114202.1	78.97	23.10
Streptomyces venezuelae ATCC-10712	CP029197.1	78.70	22.70
Streptomyces subrutilus ATCC-27467	CP023701.1	78.66	22.70
Streptomyces griseus NBC-01630	CP109279.1	78.34	22.60

Table S3. Genomic comparison among *S. olivoreticuli* YNK-FS0020, *S. olivoreticuli* ZZ-21, *S. albireticuli* MDJK11, *S. griseus* NBC-01630, and *S. venezuelae* ATCC-10712.

Features	*S. olivoreticuli* YNK-FS0020	*S. olivoreticuli* ZZ-21	*S. albireticuli* MDJK11	*S. griseus* NBC-01630	*S. venezuelae* ATCC-10712
Genome size (bp)	8,126,169	8,368,935	8,144,417	8,944,372	8,223,505
GC content (mol%)	71.42	71.23	73.00	71.50	72.50
Protein-coding genes	7105	7387	6732	7556	7276
Chromosome	2	1	1	1	1
Plasmid	1	1	0	0	0
Numbers of tRNAs	73	72	75	67	70
GenBank sequence	JBMYHM000000000	CP129915	CP021744	CP108029	CP029197
